# Impact of postpartum weight change on metabolic syndrome and its components among women with recent gestational diabetes mellitus

**DOI:** 10.1186/s12978-024-01783-4

**Published:** 2024-04-06

**Authors:** Chadakarn Phaloprakarn, Sasiwan Suthasmalee, Siriwan Tangjitgamol

**Affiliations:** 1 Department of Obstetrics and Gynecology, Faculty of Medicine Vajira Hospital, Navamindradhiraj University, 681 Samsen Road, Dusit District, Bangkok, 10300 Thailand; 2 Women’s Health Center, MedPark Hospital, Bangkok, Thailand

**Keywords:** Gestational diabetes mellitus, Metabolic risk factors, Metabolic syndrome, Postpartum weight, Weight change

## Abstract

**Background:**

While postpartum weight changes may affect the levels of metabolic parameters, the direct effects of weight changes in the postpartum period on changes in the prevalence rates of metabolic syndrome and its components remain unstudied. This study aimed to investigate the effects of postpartum weight changes between 6 weeks and 6 months on changes in the prevalence rates of metabolic syndrome and its components in women who have recently experienced gestational diabetes mellitus.

**Methods:**

This prospective cohort study included 171 postpartum women with recent gestational diabetes mellitus, who underwent serial weight and metabolic risk factor assessments at 6 weeks and 6 months postpartum. Weight changes between these time points were classified as weight loss (> 2 kg), weight stability (± 2 kg), or weight gain (> 2 kg). Metabolic syndrome comprised the following metabolic risk factors: large waist circumference, elevated blood pressure, elevated fasting plasma glucose levels, high triglyceride levels, and low high-density lipoprotein cholesterol levels.

**Results:**

Of the 171 women in our cohort, 30 women (17.5%) lost > 2 kg of body weight, while 85 (49.7%) maintained a stable weight and 56 (32.8%) gained > 2 kg. The weight loss group experienced significant changes in the prevalence rates of the following metabolic risk factors compared to the weight stability and weight gain groups: large waist circumference (% change: − 26.7 vs − 5.9 vs 5.4, respectively; *p* = 0.004), elevated fasting plasma glucose levels (% change: − 3.4 vs 18.9 vs 26.8, respectively; *p* = 0.022), and high triglyceride levels (% change: − 30.0 vs 0 vs − 7.2, respectively; *p* = 0.024). A significantly greater decrease in the prevalence of metabolic syndrome was also found in the weight loss group than in the other two groups (% change: − 20.0 vs 11.8 vs 14.2, respectively; *p* = 0.002).

**Conclusions:**

Weight changes from 6 weeks to 6 months postpartum significantly altered the prevalence rates of metabolic syndrome and its components in women with recent gestational diabetes mellitus. Early postpartum weight loss can reverse metabolic risk factors and reduce the prevalence of metabolic syndrome.

**Trial registration:**

Thai Clinical Trials Registry: Registration no. TCTR20200903001. Date of registration: September 3, 2020. Date of initial participant enrolment: September 7, 2020.

## Plain language summary

Metabolic syndrome (MetS) is a frequent diagnosis with consequences for the occurrence of cardiovascular diseases. Women with gestational diabetes mellitus (GDM) are especially vulnerable to the development of MetS. In this study, we investigated how postpartum weight changes, specifically between 6 weeks and 6 months postpartum, impact MetS and its components in women who have recently experienced GDM. The results of our study showed that women who lost > 2 kg of body weight between 6 weeks and 6 months postpartum had significant decreases in the prevalence rates of metabolic risk factors, leading to a lower prevalence of MetS, compared to women who maintained a stable weight (± 2 kg) or gained > 2 kg. Our findings suggest that such weight loss is beneficial for preventing MetS; thus, strategies should be developed to support women with GDM in achieving postpartum weight loss. These strategies may include personalized dietary counseling, exercise programs, and behavioral support tailored to the specific needs and challenges faced by this population.

## Background

Metabolic syndrome (MetS) has emerged as a global epidemic in recent decades [1], with variations in the prevalence across different ages, sexes, and ethnicities [2, 3]. Among women of reproductive age, the incidence rates of MetS and its components appear to be on the rise in recent years [4]. MetS encompasses a collection of metabolic risk factors, including abdominal obesity, elevated blood pressure (BP), elevated fasting plasma glucose (FPG) levels, high triglyceride (TG) levels, and low high-density lipoprotein cholesterol (HDL-C) levels [5, 6]. As an important risk factor for type 2 diabetes mellitus (T2DM) and cardiovascular diseases [7, 8], MetS warrants special attention.

Gestational diabetes mellitus (GDM) is a common medical complication of pregnancy. Compared to pregnant women without GDM, those with GDM often exhibit an elevated body mass index, BP, and TG level, along with a lower HDL-C level [9–11]. Furthermore, these metabolic risk factors have been observed to persist and even worsen in the postpartum period among women who have experienced GDM [12–16]. This persistence of metabolic abnormalities contributes to a higher prevalence of postpartum MetS in women with GDM compared to normoglycemic pregnant women [15, 16].

Given that abdominal obesity, as indicated by a large waist circumference (WC), is a common metabolic risk factor found after GDM [13], early postpartum weight loss may help improve a large WC and other metabolic risk factors, thereby reducing the risk of MetS. Several studies have reported significant effects of postpartum weight changes on changes in the levels of metabolic parameters such as WC, FPG, and TG in women with recent GDM [17–19]. However, no study has explored the direct effects of postpartum weight changes on changes in the prevalence rates of MetS and its components in this population.

To address this gap, we designed a longitudinal study to follow women with recent GDM after childbirth, assessing how postpartum weight changes were associated with alterations in MetS and its components over time. By examining such associations, insights can be gained into the potential benefits of weight management interventions in this population. Such findings can inform clinical practice and public health strategies aimed at reducing the long-term metabolic risks associated with GDM.

The objective of this study was to investigate the impact of weight changes occurring between 6 weeks and 6 months postpartum on changes in the prevalence rates of MetS and its components among women with recent GDM.

## Methods

### Study design and study population

This prospective cohort study was conducted between September 7, 2020, and July 31, 2023, as part of a study exploring strategies to improve the metabolic health of postpartum women with a history of GDM. The study protocol was approved by the Vajira Institutional Review Board (approval no. 017/2563) and performed in accordance with the Strengthening the Reporting of Observational Studies in Epidemiology (STROBE) guidelines. The protocol was registered with the Thai Clinical Trials Registry (registration no. TCTR20200903001).

The study population included women aged ≥ 18 years with recent GDM who delivered a live infant at the Faculty of Medicine Vajira Hospital, Bangkok, Thailand, between September 7, 2020, and January 31, 2023. Inclusion criteria were women who underwent antenatal care and GDM screening at the hospital. The exclusion criteria were pregnancy during the 6 months of study involvement, loss to follow-up, and refusal to participate.

### Sample size

Given that no study has directly explored the effects of postpartum weight changes on changes in the prevalence rates of MetS and its components among women with a history of GDM, the sample size was calculated based on data from a previous study that explored the effects of weight changes between 3 and 12 months on changes in the levels of metabolic parameters in this population [17]. Based on the findings that changes in WC among women in the weight loss, weight stability, and weight gain groups were − 5.4 ± 5.1 cm, − 1.8 ± 4.6 cm, and 1.4 ± 4.6 cm, respectively, the sample size was calculated using the data on WC changes in the weight stability and weight gain groups because this yielded the largest sample. With 80% power at a two-sided significance level of 0.05, at least 99 participants were required (33 each with weight loss, weight stability, and weight gain). Allowing for a dropout rate of 25%, the total sample size required was 132 (44 in each group).

### Institutional practices for GDM screening and diagnosis

The institutional practices for GDM screening and diagnosis have been described in detail in our previous work [20]. In brief, we followed the standard recommendation that all pregnant women undergo GDM screening using a 50-g glucose challenge test, followed by a 100-g oral glucose tolerance test (OGTT) using the Carpenter and Coustan criteria if the screening result was abnormal [21]. Blood samples were collected by phlebotomists and sent to the hospital laboratory. The initial management of women with GDM consists of dietary and lifestyle modifications, followed by insulin therapy if fasting or postprandial plasma glucose levels remain high despite dietary management [21].

After delivery, all women with GDM are scheduled for a postpartum checkup and T2DM screening using a 75-g, 2-h OGTT at 6 weeks postpartum [22].

### Participant recruitment and follow-up

Consecutive women with pregnancies complicated by GDM, who delivered between September 7, 2020, and January 31, 2023, were approached by a researcher at the postnatal ward on the second day after delivery. These women received a study outline and were invited to participate in the study. All interested women were screened for eligibility. Written informed consent was obtained from all eligible women before the beginning of the study.

At enrollment, the clinical characteristics of the participants were collected from their medical records. These included age, weight, parity, family history of diabetes mellitus, severity of GDM, and gestational age at delivery. Participants were scheduled for serial assessments of weight and metabolic risk factors at 6 weeks and 6 months postpartum.

At 6 weeks postpartum, data on breastfeeding practices were collected from the participants upon their postpartum visit. Physical examinations, including measurements of weight, WC, and BP, were performed by a nurse who had been trained to ensure standard and accurate measurements. The body weight was measured without shoes and with light clothing using the Tanita Model WB-3000 digital scale (Tanita Corporation, Tokyo, Japan). WC was measured in the horizontal plane midway between the lowest ribs and the iliac crest with the participants in a standing position. After 10 min of rest, the BP was recorded to the closest 2 mmHg using the Nova-Presameter Desk mercury sphygmomanometer (Rudolf Riester GmbH, Jungingen, Germany) with the arm supported at heart level. Three separate BP readings were taken at 1-min intervals. The average of the last two readings was used for the analysis.

Venous blood samples were drawn to determine FPG, fasting lipid, and 2-h postprandial glucose (PG) levels. Fasting lipids included total cholesterol, TG, low-density lipoprotein cholesterol, and HDL-C levels. If participants were diagnosed with T2DM, MetS, or both, they were referred to an endocrinologist for further management.

At 6 months postpartum, the same data were collected for all participants. Blood samples were collected to determine FPG, fasting hemoglobin A1c (HbA1c), and fasting lipid levels.

### Laboratory measurements

All blood samples for FPG, HbA1c, and lipid analyses were collected in the morning after overnight fasting for 12 h and sent to the hospital’s laboratory. A standard 75-g OGTT was performed to measure 2-h PG levels. Plasma glucose and lipid levels were measured using the automated analyzer Cobas c503 (Roche Diagnostics, Mannheim, Germany). HbA1c levels were measured using a Cobas c513 analyzer (Roche Diagnostics).

All devices were calibrated in-house daily and annually using external validation. Our laboratory has received National Glycohemoglobin Standardization Program certification for HbA1c assays and is traceable to the Diabetes Control and Complications Trial reference method. The HbA1c and blood chemistry analyses were approved by the Randox International Quality Assessment Scheme.

### Stratification of participants, outcome assessment, and definitions

We stratified participants into three groups according to weight changes from 6 weeks to 6 months postpartum: weight loss (> 2 kg), weight stability (± 2 kg), and weight gain (> 2 kg) [17]. The outcome measures included changes in the prevalence rates of MetS and its components.

The presence of MetS was established using the joint interim statement of the International Diabetes Federation Task Force on Epidemiology and Prevention [5]. MetS was defined as the presence of three or more of the following five metabolic components: large WC (≥ 80 cm), elevated BP (systolic BP ≥ 130 mmHg, diastolic BP ≥ 85 mmHg, or both, or treatment with antihypertensive drugs), elevated FPG levels (≥ 100 mg/dL, or treatment with antidiabetic medications), high TG levels (≥ 150 mg/dL, or treatment with drugs for elevated TG levels), and low HDL-C levels (< 50 mg/dL, or drug treatment for reduced HDL-C levels). Those with FPG levels ≥ 126 mg/dL, 2-h PG levels ≥ 200 mg/dL, or HbA1c levels ≥ 6.5% were diagnosed as having T2DM [23].

### Statistical analyses

All analyses were performed using IBM SPSS Statistics for Windows, Version 28.0 (IBM Corporation, Armonk, NY, USA). Categorical variables are presented as numbers and percentages and were compared using the chi-squared test. Continuous variables are described as means and standard deviations. Differences in the means of continuous variables between the three groups were analyzed using a one-way analysis of variance. When the overall analysis was significant, intergroup comparisons were made using the least significant difference method as a post-hoc test.

Changes in the means of variables over time (between 6 weeks and 6 months postpartum) within each group were analyzed using paired t-tests. The differences in changes between the three groups were analyzed using a one-way analysis of covariance, controlling for weight at 6 weeks postpartum and exclusive breastfeeding at 6 months postpartum, which were previously identified as factors affecting the prevalence of MetS or metabolic risk factors [17, 24]. Changes in the prevalence rates of MetS and its components over time within a group were calculated using McNemar’s test. The differences in changes between the three groups were compared using the chi-squared test. Statistical significance was defined as a two-sided *p* < 0.05.

## Results

**Fig. 1:**
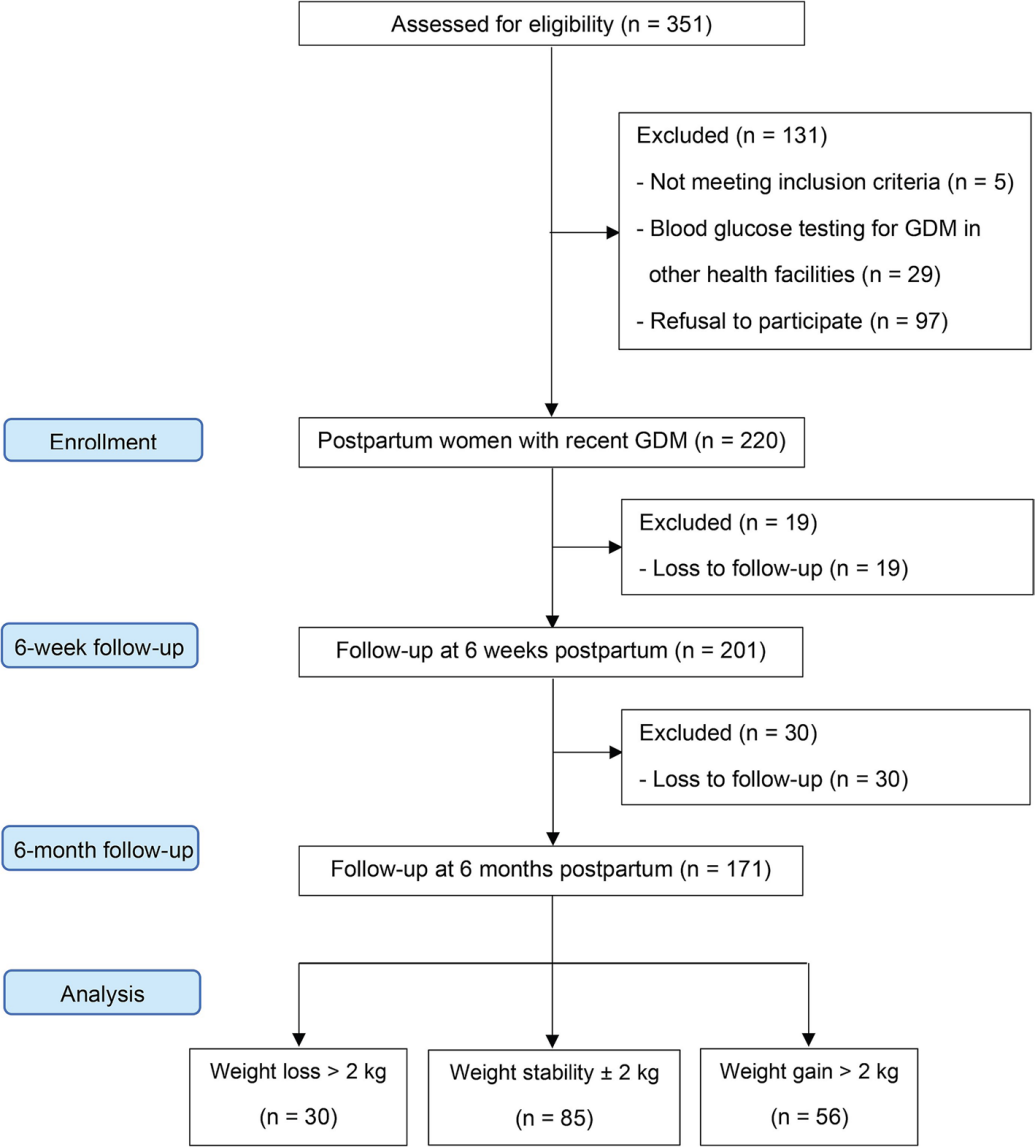
STROBE flow diagram. *GDM* gestational diabetes mellitus, *STROBE* Strengthening the Reporting of Observational Studies in Epidemiology

The study flow diagram, which is in line with the STROBE statement, is shown in Fig. 1. Of the 220 enrolled participants, 49 were excluded due to loss to follow-up before completing 6 months of participation in the study. There were no significant differences in delivery characteristics between participants who completed the study and those who were lost to follow-up (all *p* > 0.05). The 171 participants finally included were stratified into three groups: weight loss (> 2 kg; *n* = 30), weight stability (± 2 kg; *n* = 85), and weight gain (> 2 kg; *n* = 56).

### Participant characteristics

The mean age and mean weight at delivery of the 171 included participants were 32.5 ± 5.9 years and 75.4 ± 14.2 kg, respectively. Approximately 15.8% (*n* = 27) of participants had class A2 GDM. The mean gestational age at delivery was 37.8 ± 1.4 weeks. The clinical characteristics of the participants in each weight change group are shown in Table 1.

**Table 1:** Clinical characteristics of participants in each weight change group

Characteristic	Weight loss > 2 kg	Weight stability ± 2 kg	Weight gain > 2 kg	*p* value^a^
	**(*n* = 30)**	**(*n* = 85)**	**(*n* = 56)**	
At delivery				
Age (years)	31.0 ± 5.8	33.2 ± 5.7	32.4 ± 6.2	0.223
Weight (kg)	74.6 ± 13.4	73.5 ± 13.7	78.6 ± 15.1	0.104
Parity				0.675
Primiparous	11 (36.7)	39 (45.9)	25 (44.6)	
Multiparous	19 (63.3)	46 (54.1)	31 (55.4)	
Family history of diabetes mellitus	8 (26.7)	29 (34.1)	18 (32.1)	0.754
Severity of GDM				0.919
Class A1	26 (86.7)	71 (83.5)	47 (83.9)	
Class A2	4 (13.3)	14 (16.5)	9 (16.1)	
Gestational age at delivery (weeks)	38.0 ± 1.5	37.8 ± 1.5	37.8 ± 1.1	0.736
At 6 weeks postpartum				
Weight (kg)	63.6 ± 13.0	63.3 ± 12.6	67.7 ± 13.6	0.129
WC (cm)	85.2 ± 10.8	84.2 ± 10.5	86.7 ± 11.9	0.414
Systolic BP (mmHg)	121.0 ± 10.3	118.4 ± 12.1	123.0 ± 13.0	0.086
Diastolic BP (mmHg)	72.9 ± 9.2	73.4 ± 10.8	76.9 ± 9.9	0.098
FPG (mg/dL)	91.7 ± 9.3	92.6 ± 25.9	94.0 ± 16.9	0.874
2-h PG (mg/dL)	134.6 ± 39.0	134.8 ± 56.6	121.3 ± 40.0	0.244
TC (mg/dL)	218.0 ± 51.7	221.9 ± 40.5	217.9 ± 43.7	0.842
TG (mg/dL)	127.7 ± 76.5	126.0 ± 76.7	151.5 ± 78.4	0.140
LDL-C (mg/dL)	143.1 ± 48.9	151.5 ± 38.7	145.7 ± 41.8	0.557
HDL-C (mg/dL)	61.9 ± 19.4	60.5 ± 15.9	57.9 ± 14.6	0.498
At 6 months postpartum				
EBF	12 (40.0)^b^	30 (35.3)^b^	10 (17.9)	0.040
Weight (kg)	59.1 ± 12.8^b^	63.1 ± 12.6^b^	72.2 ± 14.2	< 0.001
WC (cm)	78.6 ± 10.1^b^	82.5 ± 11.5^b^	89.3 ± 12.1	< 0.001
Systolic BP (mmHg)	118.9 ± 11.7	118.8 ± 12.3	122.9 ± 12.9	0.134
Diastolic BP (mmHg)	74.1 ± 8.7	73.5 ± 10.9	76.6 ± 11.6	0.250
FPG (mg/dL)	92.8 ± 9.3	103.9 ± 36.9	105.0 ± 29.4	0.184
HbA1c (%)	5.4 ± 0.4	5.6 ± 1.0	5.7 ± 0.8	0.357
TC (mg/dL)	190.4 ± 45.1	205.7 ± 37.5	203.5 ± 36.7	0173
TG (mg/dL)	82.0 ± 50.1^b,c^	112.3 ± 68.2	130.1 ± 62.0	0.004
LDL-C (mg/dL)	125.8 ± 40.6	138.3 ± 33.9	138.6 ± 34.8	0.213
HDL-C (mg/dL)	62.2 ± 18.5^b^	58.8 ± 16.0	53.7 ± 13.4	0.040

Data are presented as the mean ± SD or n (%)

*BP* blood pressure, *EBF* exclusive breastfeeding, *FPG* fasting plasma glucose, *GDM* gestational diabetes mellitus, *HbA1c* hemoglobin A1c, *HDL-C* high-density lipoprotein cholesterol, *LDL-C* low-density lipoprotein cholesterol, *PG* postprandial glucose, *SD* standard deviation, *TC* total cholesterol, *TG* triglycerides, *WC* waist circumference

^a^Differences between groups were compared using a one-way analysis of variance or the chi-squared test

^b^*p* < 0.05, compared with the weight gain group

^c^*p* < 0.05, compared with the weight stability group

### Changes in the levels of metabolic parameters

Changes in the levels of metabolic parameters over time within and between the groups are shown in Table 2. The weight loss group showed a significantly greater decrease in WCs and a significantly smaller increase in FPG levels than the other two groups. In addition, the weight loss group showed a significantly greater decrease in TG levels than the weight stability group. The weight stability group showed a significantly greater decrease in WCs than the weight gain group. The results of the one-way analysis of covariance showed that the weight at 6 weeks postpartum and exclusive breastfeeding at 6 months postpartum have no significant effect on changes in the metabolic parameter levels.

**Table 2:** Changes in metabolic parameters within and between groups

**Metabolic parameter**	**Weight loss > 2 kg (*n* = 30)**				**Weight stability ± 2 kg (*n* = 85)**				**Weight gain > 2 kg (*n* = 56)**				
	**6 weeks**	**6 months**	**Change**	***p* value**^**a**^	**6 weeks**	**6 months**	**Change**	***p* value**^**a**^	**6 weeks**	**6 months**	**Change**	***p* value**^**a**^	***p* value**^**b**^
WC (cm)	85.2 ± 10.8	78.6 ± 10.1	− 6.6 ± 4.4^c,d^	< 0.001	84.2 ± 10.5	82.5 ± 11.5	− 1.7 ± 4.4^c^	< 0.001	86.7 ± 11.9	89.3 ± 12.1	2.6 ± 5.1	< 0.001	< 0.001
Systolic BP (mmHg)	121.0 ± 10.3	118.9 ± 11.7	− 2.1 ± 12.3	0.359	118.4 ± 12.1	118.8 ± 12.3	0.4 ± 10.8	0.757	123.0 ± 13.0	122.9 ± 12.9	− 0.1 ± 12.4	0.923	0.602
Diastolic BP (mmHg)	72.9 ± 9.2	74.1 ± 8.7	1.2 ± 11.9	0.564	73.4 ± 10.8	73.5 ± 10.9	0.1 ± 8.9	0.913	76.9 ± 9.9	76.6 ± 11.6	− 0.3 ± 9.3	0.833	0.761
FPG (mg/dL)	91.7 ± 9.3	92.8 ± 9.3	1.1 ± 7.4^c,d^	0.422	92.6 ± 25.9	103.9 ± 36.9	11.3 ± 21.6	< 0.001	94.0 ± 16.9	105.0 ± 29.4	11.0 ± 15.7	< 0.001	0.029
TG (mg/dL)	127.7 ± 76.5	82.0 ± 50.1	− 45.7 ± 49.1^d^	< 0.001	126.0 ± 76.7	112.3 ± 68.2	− 13.7 ± 60.7	0.041	151.5 ± 78.4	130.1 ± 62.0	− 21.4 ± 51.5	0.003	0.030
HDL-C (mg/dL)	61.9 ± 19.4	62.2 ± 18.5	0.3 ± 8.1	0.824	60.5 ± 15.9	58.8 ± 16.0	− 1.7 ± 10.6	0.130	57.9 ± 14.6	53.7 ± 13.4	− 4.2 ± 8.0	< 0.001	0.121

Data are presented as the mean ± SD

*BP* blood pressure, *EBF* exclusive breastfeeding, *FPG* fasting plasma glucose, *HDL*-C high-density lipoprotein cholesterol, *SD* standard deviation, *TG* triglycerides, *WC* waist circumference

^a^Changes over time within each group were analyzed using the paired t-test

^b^Changes between groups were analyzed using a one-way analysis of covariance using the weight at 6 weeks postpartum and EBF at 6 months postpartum as covariates

^c^*p* < 0.05, compared with the weight gain group

^d^*p* < 0.05, compared with the weight stability group

### Changes in the prevalence rates of metabolic risk factors

At 6 weeks postpartum, a large WC (67.3%; *n* = 115) was the most commonly detected metabolic risk factor, followed by a high TG level (32.7%; *n* = 56), low HDL-C level (28.7%; *n* = 49), elevated BP (23.4%; *n* = 40), and elevated FPG level (15.8%; *n* = 27) levels.

At 6 months postpartum, the prevalence rates of these metabolic risk factors in the order of decreasing frequency were as follows: a large WC (61.4%; *n* = 105), low HDL-C level (37.4%; *n* = 64), elevated FPG level (33.3%; *n* = 57), high TG level (25.1%; *n* = 43), and elevated BP (18.1%; *n* = 31).

Changes in the prevalence rates of metabolic risk factors over time within and between the groups are summarized in Table 3. The weight loss group had significantly greater decreases in the prevalence rates of large WC and high TG level than the other two groups. The weight loss group also showed a significantly greater decrease in the prevalence of high TG levels than the weight stability group. The changes in the prevalence rates of the five metabolic risk factors were not significantly different between the weight stability and weight gain groups.

**Table 3:** Changes in the prevalence rates of metabolic syndrome and its components within and between groups

	Weight loss > 2 kg (*n* = 30)				Weight stability ± 2 kg (*n* = 85)				Weight gain > 2 kg (*n* = 56)				
	6 weeks	6 months	Change	*p*-value^a^	6 weeks	6 months	Change	*p*-value^a^	6 weeks	6 months	Change	*p*-value^a^	*p*-value^b^
MetS^c^	11 (36.7)	5 (16.7)	− 6 (− 20.0)^d,e^	0.031	12 (14.1)	22 (25.9)	10 (11.8)	0.013	17 (30.4)	25 (44.6)	8 (14.2)	0.049	0.002
Large WC	20 (66.7)	12 (40.0)	− 8 (− 26.7)^d,e^	0.008	54 (63.5)	49 (57.6)	− 5 (− 5.9)	0.227	41 (73.2)	44 (78.6)	3 (5.4)	0.375	0.004
Elevated BP	6 (20.0)	2 (6.7)	− 4 (− 13.3)	0.219	17 (20.0)	13 (15.3)	− 4 (4.7)	0.454	17 (30.4)	16 (28.6)	− 1 (− 1.8)	1.000	0.172
Elevated FPG	5 (16.7)	4 (13.3)	− 1 (− 3.4)^d,e^	1.000	11 (12.9)	27 (31.8)	16 (18.9)	< 0.001	11 (19.6)	26 (46.4)	15 (26.8)	< 0.001	0.022
High TG	11 (36.7)	2 (6.7)	− 9 (− 30.0)^e^	0.004	22 (25.9)	22 (25.9)	0 (0)	1.000	23 (41.1)	19 (33.9)	− 4 (− 7.2)	0.454	0.024
Low HDL-C	11 (36.7)	9 (30.0)	− 2 (− 6.7)	0.687	18 (21.2)	28 (32.9)	10 (11.7)	0.064	20 (35.7)	27 (48.2)	7 (12.5)	0.092	0.389

Data are presented as n (%)

*BP* blood pressure, *FPG* fasting plasma glucose, *HDL*-C high-density lipoprotein cholesterol, *MetS* metabolic syndrome, *TG* triglycerides, *WC* waist circumference

^a^Changes over time within a group were analyzed using McNemar’s test

^b^Changes between groups were analyzed with the chi-squared test

^c^Components of MetS include (1) large WC (≥ 80 cm), (2) elevated BP (systolic BP ≥ 130 mmHg, diastolic BP ≥ 85 mmHg, or both, or treatment with antihypertensive drugs), (3) elevated FPG levels (≥ 100 mg/dL or treatment with antidiabetic medications), (4) high TG levels (≥ 150 mg/dL or treatment with drugs for elevated TG levels), and (5) low HDL-C levels (< 50 mg/dL or drug treatment for reduced HDL-C levels)

^d^*p* < 0.05, compared with the weight gain group

^e^*p* < 0.05, compared with the weight stability group

### Changes in the prevalence of MetS

The weight loss group experienced a significantly greater decrease in the prevalence of MetS than the other two groups (Table 3). Changes in the prevalence of MetS between the weight stability and weight gain groups did not differ significantly.

## Discussion

The main findings of this study were that postpartum women with recent GDM who lost > 2 kg of weight between 6 weeks and 6 months postpartum had significant improvements in metabolic parameters, leading to significantly lower rates of MetS and its components than those in women who maintained a stable weight (± 2 kg) or gained > 2 kg.

Our observation of a greater decrease in WCs and a smaller increase in FPG levels in the weight loss group than in the other two groups aligned with the results of previous studies, including women with recent GDM with a longer postpartum evaluation interval [17–19]. A study including 206 Australian women revealed significant improvements in WCs and TG levels among women who lost > 2 kg within 6 weeks to 12 months postpartum when compared to those whose weight remained stable or increased [17]. Another study involving 72 American women showed a significantly smaller increase in FPG levels in women who lost > 2 kg within 12 months postpartum than in those who maintained or gained weight [18]. Likewise, a study including 75 American women demonstrated significant correlations of weight changes between baseline (6 weeks postpartum) and 6 and 12 months postpartum with the changes in FPG and TG levels [19]. Although our study and those performed in Australia and the United States differed in terms of population characteristics (such as ethnicity and weight at delivery) and the postpartum time points at which weight changes were examined, the consistent results among these studies indicate that postpartum weight changes have the potential to impact metabolic parameters in women with a history of GDM.

To date, no study has explored how changes in postpartum weight affect the prevalence rates of MetS and its components, specifically in women with GDM. The results of our study showed that women who lost weight experienced significantly greater decreases in the prevalence rates of large WCs and high TG levels than those who maintained or gained weight. Among the two metabolic risk factors that significantly improved after weight loss, a greater reduction in the prevalence of large WCs was observed (from 66.7% to 40.0%, Δ: − 26.7%).

Moreover, we found that weight loss during the early postpartum period reduced the prevalence of MetS. The MetS prevalence decreased significantly from 36.7% at 6 weeks postpartum to 16.7% at 6 months postpartum in women who lost weight. Conversely, women with weight stability or weight gain exhibited an increase in MetS rates (from 14.1% to 25.9% and from 30.4% to 44.6%, respectively).

Our findings were consistent with those of a previous study conducted in Iran [13], which also identified a large WC as the most prevalent metabolic risk factor at 6 weeks postpartum among women with recent GDM. In contrast, a study including an American population revealed that low HDL-C levels were more common than a large WC among early postpartum women with a history of GDM [25]. These different findings may be attributed to the diverse definitions of large WCs or differences in body build among the populations studied. Our study and the study performed in Iran defined a large WC as ≥ 80 cm, whereas the study performed in the United States defined a large WC as ≥ 88 cm. Given that WC is a surrogate clinical measure of visceral adiposity, which predisposes individuals to MetS and subsequent T2DM and cardiovascular diseases, preventive measures to reduce a large WC are paramount.

Our findings underscore the metabolic implications of weight changes in the first 6 months after delivery in patients with GDM and highlight the benefits of > 2 kg postpartum weight loss in women with recent GDM. At present, there are no specific recommendations on the timing of MetS screening commencement following GDM or on the continuation of follow-up processes. The results of our study may be used to establish practice guidelines for the early detection of metabolic risk factors and MetS in women with a history of GDM. Special attention should be paid to those with weight stability or weight gain between 6 weeks and 6 months postpartum, who comprised 82.5% of this population.

Further research is needed to confirm the metabolic benefits of weight loss over a longer duration, beyond the first 6 months postpartum. Considering that postpartum women face several barriers to lifestyle modifications in terms of time constraints, childcare demands, and lack of motivation [26], interventional studies that include strategies to address these barriers should be considered to promote weight loss in the early postpartum period in women with recent GDM.

A strength of this study is its prospective serial evaluation of metabolic risk factors. In addition, the data collection procedures were standardized and reliable. All blood samples were tested using standardized assays in a single certified laboratory. Additionally, this study added new evidence that improvements in metabolic parameters resulting from weight loss in the early postpartum period could lead to subsequent decreases in the prevalence rates of MetS and its components among women with recent GDM.

Nevertheless, this study had some limitations. First, the relatively small number of women with weight loss of > 2 kg may have limited our ability to detect subtle differences in the extent and prevalence rates of some metabolic risk factors. A larger sample size would have provided more statistical power and potentially yielded more robust results. Second, this study was conducted at a specific hospital in Thailand and focused on a specific population of postpartum women with recent GDM. Therefore, the generalizability of the findings to other populations or settings may be limited. The results may not be applicable to women with different demographic characteristics or healthcare systems. Third, we excluded women who were lost to follow-up, which may have introduced selection bias. This omission could affect the representativeness of the sample and potentially influence the study's results. Fourth, our study defined a large WC as ≥ 80 cm, which may not be consistent with other definitions used in different populations or settings. This variation in defining metabolic risk factors could impact the comparability of the findings across studies and limit the generalizability of the results. Finally, this study focused on weight changes and metabolic parameters within the first 6 months postpartum. However, the long-term effects of weight loss on metabolic improvements beyond this timeframe remain unknown. It would be valuable to investigate whether the observed benefits persist or change over a longer duration.

## Conclusions

Weight changes from 6 weeks to 6 months postpartum had a significant impact on the prevalence rates of MetS and its components in women with a history of GDM. Early postpartum weight loss reversed metabolic risk factors and possibly reduced the prevalence of MetS. Given the increasing prevalence of MetS among women of reproductive age and the availability of risk-reducing strategies, we propose that all postpartum women with GDM should undergo screening for metabolic risk factors, alongside screening for T2DM.

## Data Availability

The datasets used and analyzed in this study are available from the corresponding author upon reasonable request.

## Abbreviations

BP: Blood pressure
FPG: Fasting plasma glucose
GDM: Gestational diabetes mellitus
HbA1c: Hemoglobin A1c
HDL-C: High-density lipoprotein cholesterol
MetS: Metabolic syndrome
OGTT: Oral glucose tolerance test
PG: Postprandial glucose
STROBE: Strengthening the Reporting of Observational Studies in Epidemiology
T2DM: Type 2 diabetes mellitus
TG: Triglycerides
WC: Waist circumference
